# An evolutionary case for polygyny to counter demographic collapse

**DOI:** 10.3389/fpsyg.2023.1062950

**Published:** 2023-02-06

**Authors:** Mads Larsen

**Affiliations:** Centre for Development and the Environment, University of Oslo, Oslo, Norway

**Keywords:** evolutionary literary criticism, fertility rate, marriage and family practices, mating ideology, pair-bonding, perceived sex ratio

## Abstract

Sex ratio theory suggests why mating practices have become dysfunctional in the West and other regions. Spain, Japan, and over 20 other nations are on course to have their populations halved by 2100, dramatically aging their citizenry. Experts and opinion makers warn that a demographic collapse cannot be absorbed by our current social order; Elon Musk proclaims this to be “the biggest threat to human civilization.” Statistics from the Nordic countries—the world’s most gender-equal region—indicate that subjective perceptions of the sex ratio in modern environments drive singledom and low reproduction. Scandinavia has the world’s highest occurrence of one-person households: 43–46%. In the past decade, the Norwegian fertility rate dropped from 2.0 to 1.5. Sex ratio studies suggest that women’s perception of there being few acceptable partners activates a polygynous mindset, which in prosperous, monogamous societies drives promiscuity to the detriment of pair-bonding. More than 6 million years of hominin evolution under promiscuous, polygynous, and monogamous regimes shaped mate preferences that evoke different cultural and behavioral responses as environments change. The Church’s imposition of lifelong monogamy contributed to the emergence of the modern world, but if this world’s gender-equal societies no longer motivate reproduction, being more open to polygyny could be worth considering as a means for increasing fertility. This article makes this case by exploring hominin mating from our last common ancestor with chimpanzees—through the genus *Homo’s* forager and agricultural periods—to modern Scandinavians. In the past millennium, mating practices have coevolved with the emergence of modernity, necessitating frequent cultural updates. An evolutionary analysis of Nordic works of literature illuminates the ways in which ideological narratives influence reproductive norms. The insights gleaned are considered in the context of people’s perceived sex ratio.

## 1. Introduction

Overpopulation has long been considered a grave threat to planetary health, and thus the future well-being of humanity (Parfit, 2004; Cafaro and Crist, 2012). That we now comprise more than 8 billion humans makes it harder to reduce carbon emissions and resource overuse. There are also geopolitical challenges. Having growing populations that face a perilous future can undermine local and global stability. Such concerns mark the political discourse. An even graver threat could be—against common intuition—a too rapid decrease in national populations (Bainbridge, 2009; Eberstadt, 2021). While high birth numbers in parts of the world continue to be a cause for concern (Tertilt, 2005), an increasing number of nations have experienced surprisingly drastic drops in fertility. This decades-long trend is not receiving attention commensurate to the existential nature of its potential consequences.

While low birth numbers can have a positive long-term effect on planetary health, too low fertility rates entail tremendous peril. Shrinking working-age populations can lose their ability to support relatively much larger populations of seniors. Zeihan (2022a,b) argued that Russia’s demographic decline was a main driver behind their 2022 attack on Ukraine. Strong nations will be incentivized to consolidate power as their populations dwindle. Eberstadt (2021) pointed to how economic decline is likely to change cultural psychology, motivating despondency, conflict, and anti-democratic attitudes. Psychologically, politically, and economically, we do not know how to adapt to a world with diminishing populations. A researcher warned, “It’s incredibly hard to think this through and recognize how big a thing this is; it’s extraordinary, we’ll have to reorganize societies” (Gallagher, 2020). Some opinion makers are gloomier, like Elon Musk (2021), who keeps repeating that “birth rate collapse is the biggest threat to human civilization.” Having a more energetic debate around how to manage this century’s demographic transition has become imperative.

Vollset et al. (2020) predicted that by the end of this century, 23 countries will have their population reduced by 50% or more—another 34 countries, by 25%–50%. By 2050, more than 150 countries are predicted no longer to be reproducing their numbers. As other developed countries entered into this transition, the Nordic countries long kept reproducing near replacement levels, attributed to gender equality and generous parental welfare. Some experts proposed that implementing social democratic policies could motivate other populations to reproduce. In the past decade, Nordic fertility numbers plummeted too (Campisi et al., 2022). The Norwegian fertility rate fell from 1.96 to 1.48 (Statistics Norway, 2021), Finland’s from 1.9 to 1.4, and Iceland’s from 2.2 to 1.7. The Swedish and Danish rates are 1.76 and 1.72, respectively (Grunfelder et al., 2020). The EU average has fallen to 1.5, ranging from Malta’s 1.13 to France’s 1.83 (Eurostat, 2022). This trend marks Europe, North America, and East and Southeast Asia. The lowest rates are in Taiwan, South Korea, and Singapore: 1.08–1.16 (The World Factbook, 2022).

A variety of social and economic factors are suggested to influence this unwillingness to reproduce (Weston and Parker, 2002; Grant et al., 2006; DeSantis, 2021; Eberstadt, 2021). Experts give an impression of not knowing why this is occurring or which policies could counter the demographic collapse. Their most common recommendation has been to increase immigration (Grant et al., 2006; Grunfelder et al., 2020; Vollset et al., 2020). Since many developing countries still have high fertility rates, transferring parts of their population to developed nations appeared as a viable solution. From 2000 to 2015, Norway’s immigrant population tripled (Midttun, 2018), yet the fertility rate kept falling. Today, 15% of residents are immigrants (Statistics Norway, 2022c), which increases the population, but without motivating reproduction near replacement levels. A cultural change across Europe after the 2015 migrant crisis has made continued large-scale immigration a less compelling proposition. Recent long-term-cost estimates have shown that instead of improving national finances, many groups of immigrants undermine the future viability of Western welfare states (NOU 2017:2, 2017). Eastern European and Asian cultures have been less willing to open their borders to counter low fertility (Vollset et al., 2020).

If immigration from high to low-fertility nations is unlikely to solve this challenge, other options must be explored. We also face a diminishing number of high-fertility nations from which to draw immigrants. Even today’s stark predictions could underestimate how quickly the global population could fall. Vollset et al. (2020) warned against “substantial uncertainty and diverging methodologies of estimation and forecasting.” The surprising Nordic fall in reproduction should inspire an attitude of urgency. Local scholars were caught unawares, “We’re facing China’s situation without having driven a one-child policy” (Rosenberg, 2020; my translation). This development goes against the traditional demographic transition theory whose functionalist fallacy was to assume that such things would work themselves out. Bainbridge (2009) feared that if cultural globalization continues to spread this trend, humans will end up extinct. He found no moderate, or even radical, social responses likely to work, concluding that humanity’s best hope is to become cyborgs that reproduce digitally. Such bewilderment is typical of the contemporary discourse.

Facing stakes of this magnitude, we should investigate the deeper cultural factors that influence people’s motivation to reproduce. If modern marriage and family practices (MFPs) have become dysfunctional, we should reconsider these. The prohibition of polygamy is one such MFP. Henrich (2020) accounted for how the Church’s medieval imposition of lifelong monogamy broke with antiquity’s polygynous social orders. Sexual egalitarianism offered group selection advantages to Christian cultures (Christakis, 2019)—and contributed to the West’s psychological-institutional coevolution that drove the emergence of the modern world. Imposing monogamy, especially on high-status men, was a considerable challenge. It took centuries to transition out of kinship society MFPs, which had driven high rates of polygyny in many stratified societies (Raffield et al., 2017a,b). Once the monogamous order was established, a changing environment required adaptations that kept transforming Western pair-bonding practices. Early hominins had benefited from biological evolution, developing new feelings or body shapes that matched novel mating requirements. Modern humans have been dependent on new culture. In different eras, distinct mating ideologies became hegemonic, convincing people of certain ways to think and act with regard to copulation and pair-bonding. For transmission of these norms, literature and other forms of fiction played an important part.

**Figure 1:**
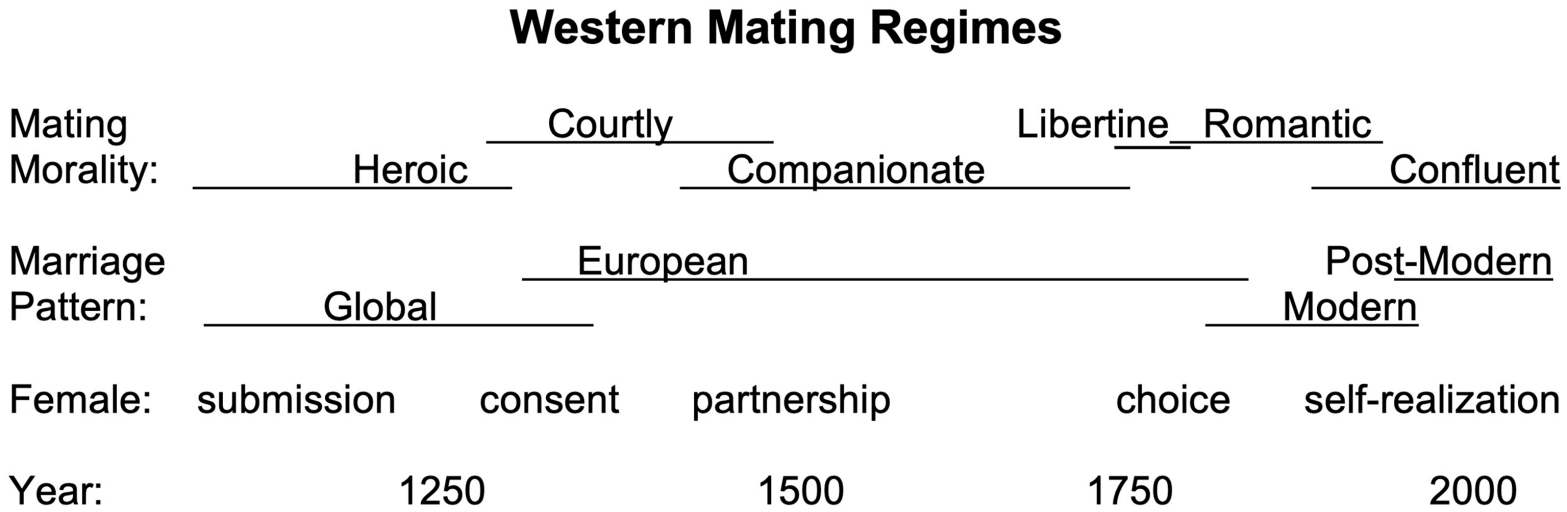
Changes in mating culture after Church MFPs dissolved Europe’s polygynous kinship societies. In this figure, I synthesize the works of Bandlien (2005), Boase (1977), Giddens (1992), Henrich (2020), Posner (1994), and Singer (1984, 1987). Copyright © 2022 by American Psychological Association. Reproduced and adapted with permission. Years correspond to transitions in the Nordic region. Each consecutive ideology empowered women as individuals. **Heroic love:** A woman should submit to the greater warrior. **Courtly love:** A man should earn a woman’s consent through chivalrous behavior and reciprocal passion. **Companionate love:** People should pair-bond informed by pragmatic concern. **Libertine love:** Pleasure-seeking through uncommitted copulation. **Romantic love:** Individuals should follow their emotions and merge with their mate. **Confluent love:** A pair-bond should last for as long as both parties benefit emotionally and/or materially. **Global marriage pattern:** A man around 30 years old marries a woman around 20, or both are around 20 and move in with his parents. **European marriage pattern:** After a period of skill and resource accumulation, a woman around 25+ establishes a new household with a slightly older man. **Modern marriage pattern:** Based on emotion and individual choice, with increasing rates of marriage as well as premarital sex. **Postmodern marriage pattern:** Low marriage rate, frequent divorce, serial monogamy, high singledom and promiscuity, and declining fertility.

The Nordic region is a good case study for the ideological development that underpins twenty-first-century MFPs. The Norse comprised the last Germanic tribes to be christened, which transpired in the indirect light of history from neighboring civilizations. Their descendants—who fled to Iceland to continue to live in a kinship society—left a testimony of their own centuries-long transition to feudalism in their medieval world literature, the sagas (Bredsdorff, 2001). Later Nordic fiction traces changes in mating ideology from antiquity’s *heroic love*, to medieval *courtly* and *companionate love*, to modern *libertine*, *romantic*, and *confluent love* (Figure 1). In this article, I argue that the tenets of confluent love contribute to today’s dysfunctional mating regime. Sacralizing self-realization, convenience, and reward, this mating ideology justifies not incurring the cost of reproduction. While earlier ideologies offered strong motivation to reproduce, confluent love makes voluntary childlessness a reasonable option.

**Figure 2:**
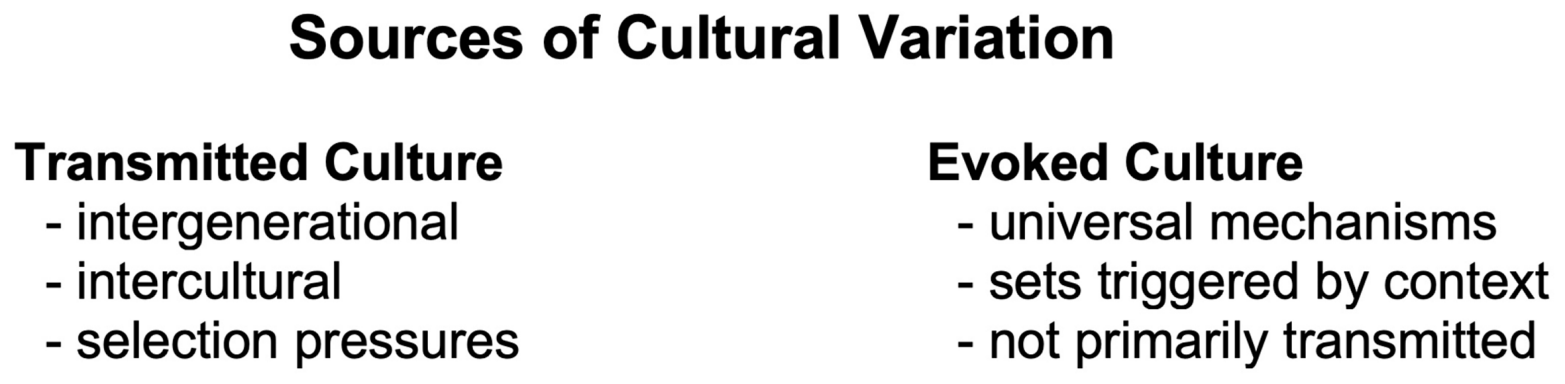
Cosmides and Tooby (1992) conceptualized that there are two sources of cultural variation in thought and behavior. The traditional explanation of within-location similarity and between-location differences is that culture is transmitted, mostly between generations but also localities. Practices are under selection pressures that determine spread. A second explanation of local similarity is that our universal, evolved information-processing mechanisms are context-dependent. When the local environment changes, certain cultural and behavioral responses are evoked based on universal predispositions—without these responses having been socially transmitted. Evoked and transmitted culture usually operate together. In matters of mating, a changed sex ratio could evoke a polygynous mating strategy in some individuals without these having been influenced by polygynous ideology or practices.

Our diverse hominin past has facilitated a range of mating strategies; local circumstances affect which are activated. The behaviors that these activated strategies motivate aggregate to what Cosmides and Tooby (1992) termed *evoked culture*. An era’s mating culture is not best understood as transmitted, or as new practices that have been subject to cultural selection; circumstances influence mating psychology in ways that evoke changes in cultural and behavioral responses (Figure 2). In parts of the world today, pressures that influence female mate preferences are, for instance, improved gender equality (World Economic Forum, 2020), increasing economic stratification (Piketty, 2022), and a marginalization of low-status men (Almås et al., 2020; Ueda et al., 2020). Nordic statistics support the idea that prosperity and gender equality can activate in women a polygynous mindset, as described by Stone, Shackelford, and Buss (Stone et al., 2007). A perception of there being few acceptable partners can motivate strategies that were adaptive under polygynous regimes. Such contexts—with a low perceived sex ratio, that is, a subjective impression of there being a scarcity of men—are in monogamous environments marked by male and female promiscuity to the detriment of pair-bonding (Schmitt, 2005; Stone et al., 2007; DeSantis, 2021; Yong et al., forthcoming). These mechanisms contribute to Scandinavia having the world’s highest occurrence of one-person households: 43%–46% (Our World in Data, 2019).

In the following, I explore the changes to hominin mating from our last common ancestor with chimpanzees to modern Scandinavians. This investigation offers insight into how *Homo sapiens* evolved for serial pair-bonding with clandestine extrapair copulation, but with remarkable flexibility to respond to circumstance (Chapais, 2008; Henrich et al., 2012; Gangestad and Grebe, 2015; Rosenthal, 2017). I examine how this flexibility is influenced by culture in my analysis of literary works from the past millennium. My biocultural approach is similar to that of Nordlund (2007) who applied an evolutionary perspective to studying forms of love in Shakespeare’s works. I consider my findings in the context of sex ratio theory, which predicts how male and female mating psychologies will respond to a perceived surplus or scarcity of potential partners. I conclude that being more open to polygyny is one of the few available means that, plausibly, could slow down the twenty-first century’s demographic collapse. Permitting polygyny would likely increase fertility, but also have negative externalities, especially for low-status men (*cf.* Costello et al., 2022). Politicians could deem such a tradeoff justifiable if they conclude that low fertility rates pose a grave enough threat. In section 4.2, I situate my argument in the context of this century’s discussions around polygamy in the West, such as a 2011 legal case in Canada in which two evolutionary scholars, Joseph Henrich and Todd Shackelford, drew opposing conclusions.

## 2. Hominin mating

The chimpanzee–human last common ancestor (CHLCA) probably lived in groups of males and females who mated promiscuously. Less than 10% of mammals pair up to breed (Kleiman, 1977; Lukas and Clutton-Brock, 2013). Among primate species, 29% do, but since chimps and bonobos do not pair-bond, neither did likely the CHLCA. Opie et al. (2013) found that there have been relatively few transitions away from pair-bonding once it has evolved. Within the assumed multi-male/multi-female groups of the CHLCA, individuals would have been free to copulate, but high-status males would have been favored for reproduction (Chapais, 2008). Such an alliance between females and high-status males is the most common reproductive strategy across many animal groups. By selecting males who outcompete others, females spread through the population whichever genes are more functional in a certain environment. The hominin starting point thus instilled in females an attraction to the most successful males.

### 2.1. From promiscuity to pair-bonding

Over the next millions of years, biparental care and pair-bonding coevolved with a growing period of offspring dependence. Alger et al. (2020) concluded that ecological change was the primary factor that enhanced the benefit of parental cooperation. Lovejoy (2009) called it a perfect storm of disparate ecological demands. Access to energy-rich, hard-to-get food enhanced male and female complementariness, making male provisioning an increasingly beneficial strategy (Kaplan et al., 2000). Chapais (2008) proposed that superior males kept harems to secure paternity certainty. That females and males cooperated to keep infants alive contributed to how, across eons, feelings evolved that motivated relationships evocative of what humans have today (Fraley et al., 2005). Like many other pair-bonding species, hominins developed a neurobiological capacity for selective social attachment facilitated by mesolimbic dopamine pathways and social neuropeptides like oxytocin and vasopressin (Johnson and Young, 2015). This transition to polygynous mating offered females copulation with successful males as well as their paternal investment.

As increasingly brainy hominins required more calories, each individual needed a larger area to forage, causing females to be more spread out (Rooker and Gavrilets, 2021). This was one of several ecological pressures that likely drove the transition from polygyny to monogamy; it became too costly to guard and provide for a multitude of females and their offspring. A male could only defend so much territory, and if this area could provide but for one nuclear family, polygyny was no longer a viable strategy. Concurrently, new tools and weapons equalized power differences, allowing inferior males more effectively to challenge mate-hoarders (Chapais, 2011). Judging by canines and sexual dimorphism, by the emergence of the genus *Homo* around 2 million years ago, monogamous pair-bonds had become the norm. We do not know for how many millions of years hominins predominantly mated under a polygynous regime, but this originary period instilled in females a bias for pair-bonding with superior males.

Gavrilets (2012) proposed that the transition to pair-bonding was driven by low-status males changing their courting strategy, and female response to such innovation. His mathematical models rejected the notion that pair-bonding could have evolved as a result of mate guarding or male provisioning. For high-status males, quantitative breeding would have remained more profitable. For low-status males, a niche opened up as the development period of offspring grew, doubling over the past 4 million years (Geary and Flinn, 2001). By offering resources in return for sexual access and exclusivity, previously marginalized males could increasingly outcompete dominant males only willing to copulate. Females faced a trade-off. They could mate with those males to whom their ancestors’ promiscuous past had made them affectively drawn—or select less compelling males who were willing to be generous.

Male provisioning and female fidelity coevolved in a self-reinforcing manner. Eventually, only very elite males would benefit from a promiscuous strategy. Gavrilets predicted that it would not make sense for females to become completely faithful; the genes of a superior male could trump access to the resources of a lower-status male. Alger et al. (2020) argued that this transition was not driven by the interplay between male provisioning and female choice, but ecological change. They still concluded the same, that the most attractive males needed not submit to trading food for sex. The process of hominin self-domestication played itself out over millions of years, until *Homo* communities comprised pairs of provisioning males and largely faithful females, in addition to a small number of polygynists and promiscuous maters.

### 2.2. A Neolithic return to polygyny

While monogamous pair-bonds became the *Homo* norm, a male bias for polygyny and promiscuity seems not to have been selected against (Chapais, 2011). Male foragers may have had limited capacity for accumulating females, but there was little pressure on them for not wanting to do so. Similarly, females concluded that provisioning low-status males mostly offered a better deal, but there was little pressure on them for not desiring a higher-status mate. Thus, women too retained a bias for polygyny in certain environments, as it can be more adaptive to be the second wife or fourth concubine of a man with abundant resources rather than to have exclusive access to a man who struggles to feed his nuclear family (Henrich, 2020). After the adoption of agriculture, these biases expressed themselves in, at times, extreme woman-hoarding. Around 90% of hunter-gatherer societies practiced polygyny, but elite foragers can rarely provide for more than four wives (Henrich et al., 2012). Agricultural surpluses and large herds allowed powerful men to build harems that could make their elite Great Ape ancestors seem prudish.

Modern Westerners may think of monogamy as being “natural,” or even a moral universal, but more than 80% of known cultures have permitted polygamy (Fisher, 2016). In some highly stratified societies, the woman-hoarding of post-Neolithic elites was so extreme that pair-bonding was mostly inaccessible for non-elite males (Raffield et al., 2017a,b). History offers numerous examples of men who accumulated harems with hundreds or thousands of women. Ancient literature and historical accounts testify to how *Homo sapiens’* polygynous bias drove war and instability. Powerful males were incentivized to compete for additional females, which motivated decades of high-risk strategies for further status elevation. Low-status males were prevented from pair-bonding if the harems of the powerful grew too large, motivating high-risk strategies to earn resources and status. These corresponding pressures on high and low-status men drove attacks on neighboring peoples as a means for copulating and pair-bonding with their women (Gottschall, 2008).

Henrich (2020) substantiated how moving away from this ancient mating morality made the modern world possible. In the fourth century, the Church began to dissolve Europe’s tribes through MFPs that entailed a dramatic break with the past. The second millennium was marked by several transitions between pair-bonding regimes and corresponding mating moralities and marriage patterns (Figure 1). This process, which we can trace through Western literature, resulted in the European Marriage Pattern (EMP) that underpinned modernity (Hajnal, 1965; Goody, 1983)—as well as the EMP’s later undoing. Fictional stories are evolutionary tools that, among much else, disseminate and forge consensus around how pair-bonding should be understood and conducted (Carroll et al., 2012, 2020). After the Icelandic sagas had examined and encouraged the feudal transition, Nordics continued to use fiction to update their mating practices as modernity emerged.

### 2.3. Heroic love

During the Viking Age (750–1050), kinship morality drove an us-versus-them mentality that justified the rape and plunder of non-kin (Henrich, 2020). While to modern minds such practices are appalling, for millennia women had to be prepared—if they wanted a chance to live and protect their children—to submit to their husband’s murderer or his allies. Scholars refer to this mating ideology as heroic love, meaning that a woman was compelled to “love,” or pair-bond with, whomever was the greater warrior (Bandlien, 2005). This regime to an extreme extent channeled mating opportunities to superior males who had little incentive not to pursue additional mates throughout life.

The sagas and contemporary accounts support that polygyny, concubinage, and sexual slavery were widespread among the Norse (Bremen, 2002; Raffield et al., 2017a,b). The narrative structure of several sagas center on the necessity for high-status men only to acquire wealth and reputation for a few years in their youth. Protagonists are frequently given 3 years to prove their mettle—before they must marry only one woman for life. These stories are set in the Saga Age—a fictionalized Viking Age—but promote the morality of Church MFPs. Viking MFPs are often omitted, whitewashed, or mentioned apologetically. In the actual Viking Age, high-status males typically would have hoarded females, but the sagas try to convince thirteenth-century Icelanders that such morality belongs to a foregone era (Larsen, 2022c).

The sagas are now commonly read to engage the thirteenth century’s pivotal question in Iceland: whether to submit to feudal rule (Bredsdorff, 2001). Icelanders had accepted Christianization in 1000, and sagas seem to have contributed to how they in 1262 accepted submission under the Norwegian king, Hakon Hakonarson. Decades earlier, this accomplished ruler had employed another genre of fiction to convince Norsemen to transition from heroic love to a very different ideology for courtship and sociality.

### 2.4. Courtly love

The Tristan Legend’s courtly branch promoted chivalrous courtship and female consent motivated by an intense affect: true love. No longer should a superior male feel entitled to a woman’s sexuality, but court her using sophisticated social skills. Copulation should be postponed until the woman feels overwhelming lust and love. Such romances emerged in twelfth-century France from which they spread across the Christian world. King Hakon commissioned a Norse version, Tristrams saga ok Ísöndar (1999 [1226]), and other romances to convince his aristocratic warriors to abandon heroic love for the only pair-bonding ideology fit for Christian men.

Baumard et al. (2022) substantiated how the emergence of courtly love was caused by high economic growth, not cultural transmission. They accounted for how across the world, romantic stories emerged after strong growth to facilitate offspring investment through a sacralization of the pair-bond. Their study of fiction from four millennia supports the idea that mating regimes should be viewed as evoked culture, meaning that universal predispositions are triggered by local circumstance (Cosmides and Tooby, 1992). Tristan romances were transmitted, but not the desire to read such literature. Baumard et al.’s rejection of institutional explanations, such as that of Henrich (2020), is a bit of a strawman. They insist that Church MFPs did not drive the transition from heroic to courtly love, but followed it. Henrich’s concept of a psychological-institutional coevolution offers a richer explanation.

To motivate Europeans to reproduce monogamously for life, courtly love exaggerated the power and duration of those emotions that had been coded into hominins to motivate pair-bonding. Such affect was connected to the one true god, as well, and to a proto-WEIRD sociality that promoted cross-cultural collaboration. This exemplifies how mating regimes and social orders coevolve. To tie the Christian world together, the interpersonal prosociality of kinship societies had to give way for the impersonal prosociality of the new mobile, educated, and transculturally inclusive European individual (Henrich, 2020). Romance knights embodied this ideal. When they acted in accordance with courtly norms and values, they would typically be rewarded with riches and a royal pair-bond. Readers were meant to follow their lead (Larsen, 2022d).

The scholarly consensus is that King Hakon succeeded. Around the end of the thirteenth century, high-status Norsemen could no longer justify rape with the heroic ideals that had been dominant for millennia. Romance ideology migrated to ballads, which seem to have triggered a popular dance craze. Instead of keeping unrelated bachelors away from unmarried women—which was common in kinship societies—communities arranged musical events that encouraged romantic mingling as an alternative to purely arranged marriages. By the mid-fourteenth century, Norse commoners too had internalized the righteousness of female consent (Bandlien, 2005).

### 2.5. Companionate love

Antiquity’s tribes appear to have kept the population size in check through murdering some of their own infants. That individuals restrained by resource access chose to limit how many mouths they had to feed in aggregate resulted in slower population growth, lessening the risk of Malthusian crises (Scheidel, 2010; Grubbs, 2013; Obladen, 2016). Such infanticide was prohibited by Church MFPs. Population growth was restricted through the EMP whose imposition of neolocal residence relegated Europeans to a period of resource accumulation before they could marry. This *nuptial valve* pushed the female marriage age up to the mid- to late 20s, shortening women’s reproductive period.

The prohibition of cousin marriage compelled people to look outside of their own community for a non-relative to wed. Courtly love had promoted such pair-bonding quests, but most people’s reality was far removed from that of courtly milieus. Dedicating years to finding one’s truelove was not an option. Companionate love was a bottom-up response from those without the resources to sacralize their own affect. This pragmatic mating ideology emphasized “the mutual responsibility of husbands and wives for running the household or farm” (Giddens, 1992). Keepings one’s children alive required fidelity and pragmatism, not indulging one’s emotions. The late medieval and early modern environments necessitated that most people resist their evolved impulses for serial pair-bonding and extrapair copulation.

All human mating regimes have had elements of companionate and romantic attitudes. I investigate not the psychological phenomenon of companionate love (Berscheid and Walster, 1978; Sternberg, 1986), but the social ideology (Giddens, 1992). At pivotal times in the past millennium, socioecological circumstances required that Europeans adapt their mating behavior, which necessitated a change in outlook that novel ideology facilitated. To convince young men to abstain from copulation and pair-bonding for more than a decade after reaching sexual maturity was a tall ask. Fiction both influenced and reflected this process.

The Unfaithful Wife (1999 [c. 1500]) offers unique insight into how a group of low-status urban apprentices experienced mating deprivation. The Danish school play combines humanistic storytelling with fifteenth-century sexual permissiveness, a combination that for historical reasons only occurred in Scandinavia (Søndergaard, 1989). A dramatic fall in population as a consequence of the Black Death had allowed a loosening of the EMP. The Church to a significant extent ignored non-sanctioned mating until new population pressures required a retightening of the EMP in the sixteenth century. *The Unfaithful Wife* dramatizes how, during this sexually permissive era, female mate preferences distributed reproductive opportunities based on status. The play encourages apprentices to work to increase their own status through prosocial means rather than to resort to behavior that could threaten group functionality (Larsen, 2022e). This pragmatic ideology remained hegemonic for another couple of centuries.

### 2.6. Romantic love

The First Sexual Revolution hit Scandinavia around 1770, a few decades after more southern Protestants had experienced the same (Johansen, 2005). This was a revolution of individual choice, of letting one’s inner feelings inform decisions of copulation and pair-bonding (Shorter, 1975) Its vanguard was the group who had been deprived of mating opportunities by the EMP. Not only had this group grown larger, but their employment would more often be found farther away from family and compensated in cash. Freer from social control, an increasing number of these young wage earners decided to live out their sexual impulses. Statistics from northwestern Europe show a dramatic rise in non-sanctioned mating; illegitimacy doubled in England, while quadrupling in France and Germany (Seccombe, 1992; Coontz, 2005). Domestic servants—a large proportion of this class—were presented as a moral vanguard when Ludvig Holberg founded modern Scandinavian drama. The region’s preeminent Enlightenment figure wrote a line of comedy plays from the 1720s on that dramatized the emerging conflict between companionate and romantic love. He anticipated the further evolution of mating ideology and practices, as well as today’s economic stratification, once people could make their own pair-bonding decisions (Larsen, 2022b).

Such agency—when the EMP’s nuptial valve was released in the mid-eighteenth century—regarded not only pair-bonding, but copulation. The transitional mating ideology of *libertine love* extolled the intoxicating pleasure that many—especially men—experienced from sex outside of committed relationships. Carl Michael Bellman composed a Swedish soundtrack to this promiscuous era. The young bourgeois turned drinking songs into world literature with his magnum opus, *Fredman’s Epistles* (Bellman, 1790), the majority of which were written around 1770. These songs embody a proto-romantic ideology that legitimizes female sexuality. A similar celebration of female lust marked fiction during the sexual permissiveness of the fifteenth century (Larsen, 2022a). Erotic agency at first resulted in many women having to deal with unplanned pregnancies on their own (Erickson, 2005). Later legislation and welfare resulted in a level of freedom that women generally had not experienced since pre-Neolithic times. Romanticism’s response to libertine dysfunction was to reconnect sex and pair-bonding through romantic love. With this term, I refer not to the universal human emotion that motivates pair-bonding (Jankowiak and Fischer, 1992), but the ideology that sacralized reproduction within a frame of lifelong monogamy. The modern era’s romantic ideals were similarly fanciful as courtly ideals had been; individuals meant for each other should merge through a pair-bond that would make its two halves whole.

In the next century, the modern novel came to distribute the ins and outs of this romantic regime. While kin had influenced marriage choice throughout humanity’s forager and agricultural phases (Apostolou, 2007, 2010), romantic love’s functionalist purpose was to empower people to make their own decisions. That hegemonic ideology prescribed individual choice resulted in more people giving in to their evolved mating impulses. Fortuitously, a more productive environment, along with emigration opportunities, prevented a Malthusian crisis. Romanticism too exaggerated the power and duration of pair-bonding affect, but this had an adaptive function: to convince young lovers to prioritize reproduction. The nineteenth century’s population explosion suggests that romantic love was a compelling proposition.

This ideological narrative began unraveling partially as a consequence of the Darwinian revolution. The idea of humans as evolved apes inspired a literary movement consisting of Henrik Ibsen, August Strindberg, and other Nordic playwrights and novelists who applied evolutionary perspectives to untangle humanity’s true pair-bonding nature (Larsen, 2021, 2023b). Their insights lay the foundation for twentieth-century gender equality and social democratic governance, but also the demythologized mating ideology that grew hegemonic after the Second Sexual Revolution of the 1960s (Larsen, 2023a)—which entailed a reorientation toward “the hard sexual core, thinking eroticism most precious in what human relationships have to offer” (Shorter, 1975).

### 2.7. Confluent love

The West’s present-day pair-bonding beliefs sacralize convenience and self-realization. People are viewed as reward-maximizing individuals who should pair up for as long as their emotions or pragmatic concern motivate them to do so (confluence: coming together). When a pair-bond no longer is beneficial, one moves on to singledom or the next bond; *Homo sapiens* has returned to the serial pair-bonding for which it evolved. Extrapair copulation still is mostly socially unacceptable—as it also was in most forager communities (Chapais, 2011)—but sanctions tend to be less severe than in previous centuries. This morality is conveyed through romantic comedies and other forms of fiction, as well as other media, throughout the West and other parts of world.

In the Nordic countries, generous welfare frees more women to break out of, or avoid, burdensome bonds (Trägårdh, 1997). Similar to the way in which early hominin females could make do without paternal care, modern women can raise children on their own. Buss (2016) wrote that with long maternity leave, subsidized daycare, and other forms of support, Nordic “taxpayers effectively provide women with what partners otherwise would.” In Norway, social democratic governance on average transfers $1.2 million more to each woman over a lifetime than she pays in tax. The average man pays more in tax than he receives in benefits (Statistics Norway, 2022d; national oil revenue also counted as tax). Women’s economic independence affects male cognition; mate guarding has become less of an imperative—Swedish men now value female chastity at a worldwide low (Buss, 1989). This social order facilitates Scandinavia’s high female labor force participation, further empowering women (Bovino and Gold, 2017)—and affecting their mating cognition too.

Nordic women being less dependent on male provisioning influences how their mate preferences play out. From 1985 to 2012, the number of Norwegian men who failed to reproduce by age 45 increased from 14% to 23% (Amundsen, 2014). Three times as many men as women suffer involuntary childlessness (Håkonsen and Krekling, 2017). Experts attribute this inequality to women’s recycling of high-value mates (Jensen and Østby, 2014)—which can be viewed as a form of temporal polygyny. Norwegian men with high salaries have a 90% chance of being pair-bonded by age 40—those with low salaries, a 40% chance (Almås et al., 2020). Danes experience a similar marginalization: 45% of low-skilled men live alone (Forum for Mænds Sundhed, 2017).

American men also face stronger selection pressures. Over the past two decades, past-year sexual inactivity among young men rose from 19% to 31%, a trend that disproportionally affects those with low income (Ueda et al., 2020). Another survey indicated that from 2008 to 2018, virginity among American men under age 30 rose from around 8% to 27% (Ingraham, 2019). A variety of male-driven factors could contribute to this sexual marginalization of some men, such as decreased testosterone levels (Travison et al., 2007), porn use (Park et al., 2018), and the use of digital media (Sansone et al., 2017). In terms of female-driven factors, sex ratio studies suggest that to understand why women to an increasing extent discriminate against low-status males, we must consider evolved mate preference mechanisms.

## 3. Sex ratio theory

Our evolutionary past informs why one of the greatest psychological sex differences is between male and female mating preferences. That hominin females grew dependent on male provisioning motivates a greater interest in cues of commitment and resource provisioning ability (Buss, 2016; Luoto, 2019; Walter et al., 2020). That males can strengthen their genetic legacy through promiscuous or polygynous mating motivates a greater preference for partner variety (Clark and Hatfield, 1989; Buss and Schmitt, 1993). How these preferences inform the respective sexes’ mating strategies is influenced by how many potential partners there are in their environment. Sex ratio is a term for the relative number of men to women in a given population. A high ratio involves an oversupply of men. A low ratio entails that there are more women. War, violence, emigration, urbanization, selective infanticide, and gender over-mortality are among the factors that can skew the ratio.

### 3.1. Subjective perceptions of scarcity

A weakness in sex ratio studies has been their focus on the adult sex ratio (ASR: men and women of reproductive age). The operational sex ratio excludes those in relationships (in monogamous environments, the OSR corresponds with the ASR). However, for women to perceive a man to be “operational”—that is, attractive enough for mating—more is required than him merely being single. Filser and Preetz (2021) found only a weak correlation between the ASR and the subjective mating market experience of individuals. Breaking numbers down into narrower age brackets helped, but to establish people’s actual perception of mate availability ratios—Filser and Preetz concluded—studies must consider male and female preferences for hyper or hypogamy, that is, respectively, to partner with someone of higher or lower status. The concept of perceived sex ratio (PSR) engages how socioecological circumstances influence mate preferences (Hahn et al., 2014).

As our exploration of hominin mating showed, our female ancestors had a bias for high-status males, a bias that has remained with our lineage (Buss and Barnes, 1986; Buss, 2016). The later forager ecology drove them to accept pair-bonds with low-status males (Gavrilets, 2012)—as did Church MFPs (Henrich, 2020). Companionate love encouraged women to mate with those men who were available—irrespective of affective motivation. This was highly adaptive in environments more impoverished than those of today’s developed world, which to a greater extent free women from needing male provisioning (Buss, 1989).

An adherence to confluent love in prosperous, gender-equal nations with generous welfare, like the Nordic ones (World Economic Forum, 2020), seems to trigger in women a low-ratio response, a shift in mate preferences that would have been more adaptive under a polygynous social order. Even if a woman faces an ASR of 1:1, her psychology will respond to the PSR, the prevalence of men whom she finds attractive enough for mating. Financially empowered women are likely to perceive the pool of potential mates to be smaller compared to when securing the provisions of a low-status male was of crucial importance (DeSantis, 2021). The Nordic experience of the past decades, as we saw in statistics and expert analyses from the region, supports such a hypothesis.

### 3.2. The alternative hypothesis

Using social exchange theory, Guttentag and Secord (1983) popularized research on how skewed ratios affect men’s and women’s mating strategies. Pedersen (1991) introduced evolutionary theory to the emerging field of sex ratio studies. He predicted that intrasex mate competition would be informed by the preferences of the scarce sex. If part of a surplus, men will act more like women want them to—and vice versa (Schacht and Borgerhoff Mulder, 2015). This mechanism, in high-ratio environments, drives fidelity and investment in the pair-bond—when the ratio is low, in promiscuity and relationship instability (Secord, 1983; South, 1995; Schmitt, 2005). Biologists predicted that since increased competition would reduce an individual’s chance at success, alternative courses of action could offer greater benefit. For instance, a man facing scarcity could increase his mating success by offering more care to his mate rather than to compete for additional mates. Conversely, when a man faces a surplus, he will be incentivized to compete for additional mates since this has become more likely to pay off (Kokko and Jennions, 2008).

These insights inform the Alternative Sex Ratio Mate Preference Shifts Hypothesis, a response to the Classical Hypothesis, which predicted that both sexes, when facing a deficit of potential partners, will lower their standards to increase their chance of attracting a mate. Similarly, the Demographic Opportunity Thesis had proposed that both sexes, if facing a surplus, will engage in more short and long-term mating (Adkins et al., 2015). Empirical research went against such a universalist understanding (Schmitt, 2005). The Alternative Hypothesis posits that men and women will respond to a low-ratio context—that is, a surplus of women—in ways that can appear counter-intuitive. Instead of raising their standards to attract a higher-value partner, men would lower their standards to have more promiscuous sex. Instead of lowering their standards to attract a mate, women would raise their standards to avoid being deceived by men who seek short-term mating (Stone et al., 2007).

The Classical Hypothesis received partial empirical support in that men will generally express lower standards when facing a scarcity. Humans practice assortative mating (Buss and Barnes, 1986), but with a high sex ratio, men’s reduced choosiness gives women a higher chance of marrying up (Albrecht et al., 1997; Pollet and Nettle, 2008). Women’s response to scarcity was more complex. They compete more fiercely and do so by catering to male mate preferences. Women permit more uncommitted sex (Schmitt, 2005). They signal promiscuity, for instance, by wearing shorter skirts (Barber, 1999). A novel expression of such female–female competition, informed by male preferences, is that when high income inequality reduces the proportion of attractive bachelors, women post more sexualized selfies (Borgerhoff Mulder, 2018). Early pregnancy is another competitive means. In low-ratio contexts, women appear to compete for males through teenage pregnancies and pregnancies outside of marriage (South and Trent, 1988; Barber, 2000, 2001; Chipman and Morrison, 2013).

### 3.3. A polygynous mindset

In spite of these behavioral adaptations, women do not act in line with what the Classical Hypothesis predicts; when facing scarcity, they typically do not express lower standards for a long-term mate. Instead of marrying down, they often prefer singledom (Lichter et al., 1995). Stone et al. (2007) began by hypothesizing that when women face an environment in which men to a greater extent pursue short-term mating—because female abundance incentivizes them to do so—women have an evolved defense mechanism that makes them adopt higher standards. Sensing that men of similar mate value are now more likely only to want short-term mating, women avoid deception by mentally excluding such men from their pool of potential mates. Such a mechanism, Stone, Shackelford, and Buss suggested, may have contributed to women’s greater reproductive success in our evolutionary past.

In some low-ratio environments, women’s actual marital decisions do entail a lowering of standards. Their psychology may respond to protect them from men with short-term intentions, but when offered long-term commitment, some women choose to marry men who are less educated, or older, than the women would have found acceptable in a more gender-even environment (Spanier and Glick, 1980). Such a lowering of standards in terms of actual behavior happens, but not frequently enough to support social exchange theory, which predicts that increased male bargaining power generally should motivate women to marry down. One study found that in American cities with a female surplus, women are more likely to remain unmarried than to marry a low-status man (Lichter et al., 1995). An Asian study found that women—and men—are increasingly choosing to remain single rather than to marry someone they perceive to have insufficient status (Yong et al., forthcoming).

This complexity made Stone, Shackelford, and Buss reconsider their hypothesis. Women’s increased promiscuity in low-ratio environments appeared to be an adaptation to men’s greater preference for copulation without commitment. DeSantis (2021) concluded that women simply switch to a short-term strategy. But why would women opt to have more promiscuous sex, yet also raise their standards to counter the short-term strategies of men? Unfortunately, Stone et al.’s data did not inform them of whether women had sex with lower, similar, or higher-value men—or whether men changed their mate preferences with regard to short-term partners. They just knew that men and women—overall—had more uncommitted sex in low-ratio contexts. That women become more promiscuous, while also raising their mate standards—Stone, Shackelford, and Buss proposed—could reflect the activation of a polygynous mindset.

## 4. Discussion

In pre-modern polygynous societies, it may have been adaptive for unmarried women to be more responsive to the advances of higher-status males than it is under a monogamous regime with assortative mating. Increased promiscuity, directed at prosperous targets, could be a strategy that evolved to attract high-value polygynists rather than the low-value monogamists whose evolutionary niche had centered on partner exclusivity. That low-status women in low-ratio contexts compete for men through early and extrapair pregnancy supports this hypothesis (Barber, 2000; Chipman and Morrison, 2013)—although under monogamous regimes, such a strategy is less likely to pay off in terms of pair-bonding. Similar support is offered by Nordic and other Western statistics that report high singledom and promiscuity in combination with an increasing sexual marginalization of low-status men (Almås et al., 2020; Ueda et al., 2020).

### 4.1. Postmodern marriage pattern

Over the past century and a half, educational and professional opportunities have empowered women. While this has been of tremendous benefit in many regards—for women and society overall—in terms of pair-bonding, there have been negative externalities. People generally express a desire for relationships and tend to be happier when coupled up (Argyle, 1999; Diener et al., 2000; Grover and Helliwell, 2019)—yet singledom has become more prevalent (Fry and Parker, 2021). Over the past four decades in Norway, the proportion of people not in established relationships has increased from 24% to 33% (Bergløff, 2021). An important driver of this development appears to be how female mate preferences disincentivize women from settling for the increasing number of men who now have relatively lower status—and less crucial provisioning to offer (Buss, 2016; Brooks et al., 2022). This can be viewed as a low-PSR effect. The ASR may be even, but female aversion to hypogamy on long-term markets, and a propensity for hypergamy on short-term markets (Bereczkei et al., 1997; Buss and Schmitt, 2011), channel sexual opportunities to high-value men, while reducing the prevalence of long-term pair-bonds and reproduction. In previous eras, high promiscuity could boost fertility. With modern contraception and abortions, women can choose when to reproduce—and with whom not to do so. If women find themselves without a sufficiently attractive man willing to commit, their desire to copulate can be fulfilled without incurring the cost of reproduction.

These mechanisms in combination with a belief in confluent love have contributed to a postmodern marriage pattern evocative—at least age-wise—of the EMP that Western nations transitioned out of under the influence of romantic love. The EMP had driven women’s age at first marriage up to the mid- to late 20s. With modern mating ideologies, this number fell, in particular after WWII (Coontz, 2005). In 1974, the typical Norwegian woman was 23 years old when she married. In 2020, she was 34 (Statistics Norway, 2015, 2022a)—although her first birth was at 30 (Statistics Norway, 2022b). Over this period, her fertility rate fell from 2.13 to 1.48 (Statistics Norway, 2022e). Restricting population growth—which during antiquity was achieved through infanticide, and later through the EMP—is now achieved too effectively through birth control, confluent love, and the imposition of monogamy on women whose mate preferences disincentive them from pair-bonding with a large proportion of the available men.

### 4.2. Twenty-first-century polygyny

**Figure 3:**
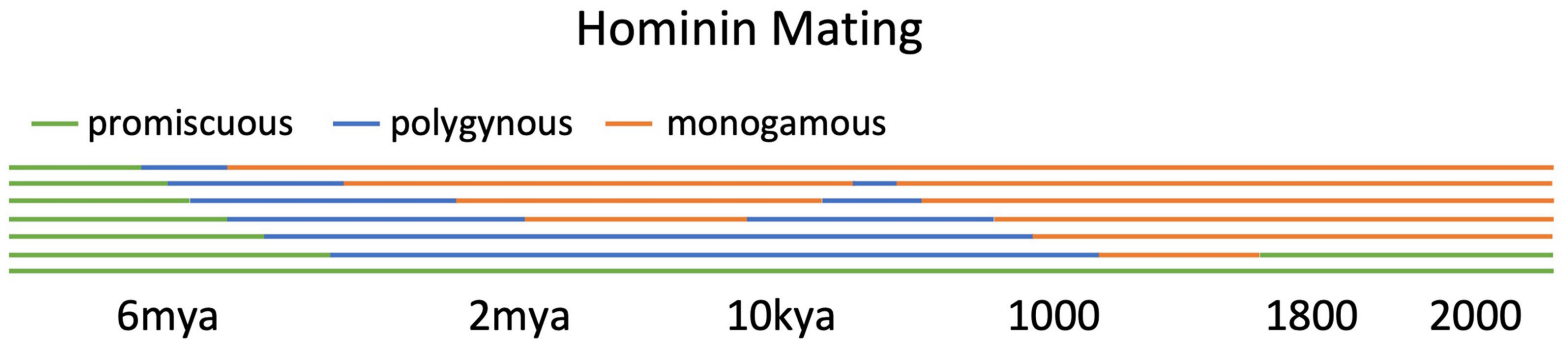
A possible evolution of mating practices. Global pattern until the Middle Ages, then Western. This figure synthesizes the works of Ågren and Erickson (2005), Chapais (2008, 2011), Fisher (2016), Henrich (2020), Karmin et al. (2015), Opie et al. (2013), Seccombe (1992), and Zeng et al. (2018).

While polygyny appears to have been adaptive and widely practiced among early hominins and many *Homo* agriculturalists (Figure 3), this pair-bonding regime was somewhat demonized in the modern West—and not without reason. Nineteenth-century Americans compared polygyny to slavery, terming them “the twin relics of barbarism” (Gordon, 1995). In the pre-modern environment, polygyny had commoditized women, motivated violence, instability, and war, and driven a high-testosterone zero-sum mindset that reduced in-group cooperation and trust (Ember et al., 2007; Witte, 2015; Raffield et al., 2017a,b; Henrich, 2020). Today, sub-Saharan polygyny has negative externalities such as stunted economic growth and unsustainably high fertility (Fenske, 2015). Tertilt (2005) estimated that ending polygyny could increase African per-capita output by 170% and decrease fertility by 40%. The German economist is supported by historical evidence. Church MFPs empowered females, underpinned economic growth, reduced Western fertility, and offered more mating opportunities for low-status men. Ending polygyny was part of what made the modern world emerge (Christakis, 2019; Henrich, 2020).

Today’s Western environment is different from those of antiquity or present-day Africa. To what extent reintroducing polygyny must come with the same downsides was the crux of a 2011 legal case in British Columbia. Joseph Henrich and Todd Shackelford wrote contradicting reports. Henrich warned against legalizing polygamy, thinking this would drive unmarried men to crime. Women would marry younger, age gaps would increase, husbands would control their wives more, and offspring would receive less paternal investment. GDP *per capita* would decrease due to an increase in fertility and men’s greater emphasis on obtaining additional wives (Henrich, 2010). Shackelford pointed to how all pair-bonding regimes have negative correlates and apparent consequences, citing how monogamy contributes to jealousy and violence. He rejected Henrich’s extrapolation from the available data without accounting “for possible cultural and contextual differences in polygynous relationships” (BCSC 1588, 2011).

Both agreed that our evolved psychology biases people to polygyny, but Henrich thought that by legalizing such pair-bonds, we risked a rapid transformation of Western societies. In repeated surveys of his undergrads, he had found that 70% of females would prefer being the second wife of a billionaire rather than the exclusive wife of an average man (BCSC 1588, 2011). Since women are so drawn to marrying up, polygyny may not remain a niche mating strategy, but spread to the majority population. Polygynous immigration would be another factor, should the Canadian court allow the practice. Considering how salient such pair-bonding is, these foreigners could inspire natives to copy them. Henrich cited how a Hindu actor in India converted to Islam to marry more women. Once high-status men turn polygynous, such practices could be socially legitimized among lower-status populations. Henrich referred to how polygyny purportedly was spreading among some Muslim African-American men. Population segments who adopt this practice would grow more rapidly, stated Henrich, because “the fertility is always higher in polygynous communities.” Shackelford ceded that it was “plausible” that polygyny could spread across North American populations, but concluded that this “seems terribly, terribly unlikely” (BCSC 1588, 2011).

The Canadian court upheld that polygamous practices—whether through formal marriage or not—should be punishable by up to 5 years in prison. They adopted the position of political scientist Rose McDermott: that the harms of polygyny are universal, irrespective of “regional, religious, or cultural context” (BCSC 1588, 2011). The US Supreme Court facilitated a similar outcome in 2017, declining a case from the stars of the polygynous reality show Sister Wives (2010). They had won a 2013 case in Utah, which had been reversed in 2016. The popularity of this 17-season TV show, and these court cases, attest to a cultural climate that is becoming more positive to polygamy. From 2003 to 2022, the number of Americans who think marrying multiple people is morally acceptable showed a steady increase from 7% to 23% (Gallup, 2022). The proportion of Canadians who support decriminalization has risen to 36% (Ipsos, 2018). In the 2020s, several American cities have recognized polygamous domestic partnerships (Economist, 2023). A variety of scholars encourage reconsideration, emphasizing that modern polygamy can empower women as well as inspire pride and belonging (Calder and Beaman, 2014). DeBoer (2015) argued that, after the legalization of same-sex marriage, group marriage is “the next horizon of social liberalism.” Mendelson (2022) asked understanding for many women preferring “to be the plural wife of a fine man rather than the single mate of an ordinary or inferior one.” Nussbaum (2008) wanted the state to “leave the field of intimate sexual choice to a regime of private contractual arrangement. If polygamy turns out to be a bad idea, it will not survive the test of free choice over time.”

In the contemporary discourse, challenges to serial monogamy are increasingly voiced from a perspective of “polyamory,” a trendier, more inclusive label for multi-partner relationships (Strassberg, 2003). Considering the history of hominin mating, the low prevalence of polyandry (around 1% of cultures; Murdock and White, 1969), and the environments in which this occurred (very impoverished ones), polyamory would seem likely to result predominantly in polygynous pair-bonds. Sexually, perhaps our present era’s greater permissiveness would facilitate more inter-wife and extrapair copulation, but it seems unlikely that many women would build harems.

To what extent contemporary maters would replace serial monogamy—what Shackelford termed “effective polygyny” (BCSC 1588, 2011)—with actual polygyny is hard to predict, as Shackelford’s strong disagreement with Henrich testified to. The two evolutionary scholars also hotly disputed to what extent historical downsides will repeat themselves if polygyny is reintroduced. McDermott’s support of Henrich convinced the court, but her universalization of polygynous harm seems too bold. A practice that appears to have been hegemonic for millions of years, and then for millennia, should hardly be written off as something that must be a net negative for human well-being. That polygyny had adverse consequences in antiquity, and other contexts, must not mean that monogamy would have been a superior regime. WEIRD-centrism likely contributes to how we condemn polygyny in Africa too; not all evidence aligns with such a position (Lawson et al., 2015). The least avoidable cost is perhaps that reintroducing polygyny would incentivize men to increase their mating effort at the expense of paternal investment, but it is hard to predict the consequences of such a reallocation. A reduction in parental overinvestment, so-called *helicopter parenting*, could even be beneficial (Lukianoff and Haidt, 2018). Polygyny may come with serious negative externalities, but what Henrich cited as one of them, “higher fertility rates,” could be of such benefit to nations facing a demographic collapse that the outcome would still be net positive.

### 4.3. Responses to polygyny

Among evolutionary scholars, there is little disagreement with regard to polygyny being a compelling proposition for *Homo sapiens* in many environments. Henrich and Shackelford mostly disagreed on whether permitting polygamy would be a social net benefit. That many individuals are drawn to such mating does not let us conclude that permitting multiple wives is the prudent thing to do. Potential positives would have to be weighed against potential negatives. For instance, a graver discrimination of low-status men could have adverse consequences for their well-being (*cf.* Costello et al., 2022). The evolutionary sciences can point us to contexts for effective intervention, but only to inform the political debate that should precede decision. Researchers have a particular responsibility in this regard. Conveying a deeper understanding of human mating, informed by our evolutionary past, can help people better understand their own conflicting impulses. This can be of particular value in matters of mating, which tend to rouse strong emotion, but we must resist the impulse to view evolutionary insights as settled science. That social complexity makes outcomes hard to predict should also warrant caution with regard to suggesting policy (Segerstråle, 2000; Alcock, 2001; Laland and Brown, 2011; IJzerman et al., 2020).

An evolutionary approach can help us make sense of contradictory mating preferences. Some women may reject polygyny because they would prefer exclusive access to a high-value mate, but that math does not add up, especially in our stratified modernity. With today’s enforced monogamy, more women are relegated to mate with low-status men, share higher-status men in temporal succession, or be single. Statistics show that women are increasingly unwilling to copulate or pair-bond with less attractive mates. Although women desire relationships, as they have gained equality, their standards have increased. Today’s rising economic stratification motivates further discrimination of certain men. With improved gender equality, women sorted away the poorest men. Rising economic inequity makes women exclude those men who are just below average (Brooks et al., 2022).

Being a high-value man in the modern environment is about more than financial capital. Women generally want men with high education and status, financial success, greater intelligence, a tall stature, independence, and self-confidence (Buss, 2016; DeSantis, 2021). Those unable to attract such a man may forego pair-bonding and reproduction to prioritize other sources of fulfillment, such as a rewarding career or financial independence (Sng and Ackerman, 2020). Other contemporary pressures also affect the equation. Many women cite a lack of social support or fears for the future as reasons for not having kids (Blackstone, 2019; Cronin, 2019). The poorer women experience themselves to be doing on the mating market, the more they emphasize the importance of other life goals, like individualistic pursuits, avoiding social conflict, and having time for oneself (Apostolou and Christoforou, 2022). Such goals are sacralized by confluent love whose core tenet is individualistic self-realization.

Many women who reproduce share men in succession, a form of temporal polygyny that breaks families apart. Henrich pointed to how high-status men “divorce the older wife in order to marry a younger wife, and in a polygynous society they would just add a younger wife. It’s a lot more convenient; you can still live with your children” (BCSC 1588, 2011). He said this to warn against how popular polygyny could become. Considering how intact families promote well-being (Amato, 2000), Henrich’s point could be used to argue against his conclusion. In 2011, he seemed not to fully appreciate how singledom and low reproduction have come to plague modern societies. For those 45% of low-skilled Danish men who live alone, their life expectancy is 7 years shorter than that of pair-bonded men (Forum for Mænds Sundhed, 2017). Norwegian men who are single and childless at age 50 are twice as likely to die early. From 1980 to 2020, early death mortality for unmarried men—and women—rose from being 20% to 80% higher than that of married people. Researchers do not know why this mortality stratification is occurring (Bergløff, 2021).

How the hegemonic ideological narratives compel so many to reject non-monogamous mating arrangements contributes to unwarranted stigmatization of otherwise functional relationships and undermines our understanding of feasible alternatives (Yong and Li, 2022). With increasingly negative externalities from today’s regime of serial monogamy, reconsidering polygyny is becoming more of an imperative. We can do this without callously disregarding the plight of low-status men. We should sympathize with men who suffer women’s increasing discrimination—not only because this the humane thing to do, but out of self-interest. Historically, and in parts of the world today, such men have caused harm and disruption. The *young male syndrome* has been connected to social instability, crime, violence against women, and aggressive and risk-taking behaviors (Blake and Brooks, 2022). An important difference in today’s modern, monogamous societies is that these men are already being deprived of mating opportunities. High rates of singlehood (Bergløff, 2021; Fry and Parker, 2021), and increasing sexual inactivity among low-status men (Ingraham, 2019; Ueda et al., 2020), suggest that permitting polygyny would be less detrimental to the well-being of undesirable men. They have already been marginalized by a *de facto* polygynous mating regime facilitated by serial monogamy, liberal attitudes toward uncommitted sex, and the market efficiencies created by online dating apps (Blake and Brooks, 2022).

Legally permitting women to share high-status men would therefore entail less downside for overall well-being than if the medieval or modern marriage patterns were intact. One of the EMP’s defining features was its extraordinarily high percentage of never-married women: around 10% (Erickson, 2005). That 33% of today’s Norwegians are not in established relationships makes for a radically different environment (Bergløff, 2021). Returning to the 1950s’ sexually egalitarian environment of near-universal marriage seems not to be an alternative (Coontz, 2005). Mating stratification seems more likely to increase. Women circumvent the current regime through a variety of informal practices, for instance, “sugar-dating” (Recio, 2021). “Incel culture” testifies to how low-value men already are feeling left behind (Hoffman et al., 2020; Costello et al., 2022). Online forums are filled with depressed, often misogynistic testimonies from the increasing number of men who are unable to compete on today’s mating markets. Some go further; in the period 2014–2018, incels killed 50 people in North America and Europe (Blake and Brooks, 2022). As long as we are unable to prevent their increasing marginalization—and economic stratification in general (Harris, 2017; Yang, 2018; Sandel, 2020)—adapting to current realities should be on the table.

If low-status men are unlikely to experience an increase in mating opportunities, other measures could reduce their ill-being more than upholding the prohibition against polygamy. Lessening the cultural condemnation they suffer could be a starting point. An evolutionary approach to women’s mate preferences makes it clearer why incels should not primarily be understood as “self-absorbed and pathetic” (Blake and Brooks, 2022). Socially permitting that these men ideologically opt out of pair-bonding—exemplified by the Men Going Their Own Way movement (Jones et al., 2021)—could reduce their pain and resistance, and perhaps misogyny (Grinde, 2021, 2022). Female mate preferences may drive our era’s reduction in pair-bonding, but women are no more in control of their evolved psychology than men are of theirs. More openness around how our mating impulses play out could perhaps inspire a greater understanding for why enforced monogamy works less well in prosperous, gender-equal societies. Confluent love already justifies polygyny, I would argue, as long as people understand how convenient and reward-maximizing such mating could be. Thinking that two people meant for each other should merge for life is a remnant of romantic love. The increasing North American acceptance of polygamy supports this position (Ipsos, 2018; Gallup, 2022).

### 4.4. High-ratio prosociality

I argue not that many nations are headed for a demographic collapse because they enforce monogamy. This development is driven by complex causality. I argue that permitting polygamy could be the most effective means at our disposal for countering a too rapid decline in fertility. If (1) we conclude that quick depopulation is likely to have grave consequences for human well-being (Gallagher, 2020; Eberstadt, 2021; Musk, 2021), and (2) we are unable to find other effective means for increasing fertility (Bainbridge, 2009; DeSantis, 2021), we should give polygyny serious consideration. Whether we then choose to permit such pair-bonds is a political question.

Even if permitting polygamy on its own did not fully solve the problem of population decline, it could have additional benefits. An increase in pair-bonding would be positive, facilitated by letting women share high-value mates and preventing breakups driven by serial monogamy. Reducing singledom could have favorable side effects. Creating a higher-PSR environment, a perception of women being scarcer, could have a line of prosocial implications that might outweigh negative externalities from reintroducing polygyny (*cf.* Costello et al., 2022). We saw how when women perceive that there are few attractive bachelors, they seem to adopt a polygynous mindset that in monogamous environments drive promiscuity to the detriment of pair-bonding. Men could also perceive a 1:1 ASR to be a low-ratio context. Filser and Preetz (2021) found only a weak correlation between the ASR and the PSR. We lack solid data for this, but today’s high prevalence of single women could be triggering in men a dysfunctional mindset of female abundance. Stone et al. (2007) found that in low-ratio contexts, men in general raise their standards for long-term partners. Low-status men seem to respond to twenty-first-century promiscuity and singledom—even if copulation and pair-bonding opportunities mostly are channeled to high-status men—with a choosiness that makes it even harder for them to find a mate (Yong et al., forthcoming). Polygynous competition could make such low-ratio delusions harder to maintain.

Sex ratio studies have shown that low-ratio environments motivate promiscuity, dating deception, illegitimate births (Schmitt, 2005), teenage pregnancies (Barber, 2001), less stable marriages, and more singledom (Secord, 1983; Pedersen, 1991; South, 1995). Facing a surplus of women, men cohabitate less and have more dating partners (Warner et al., 2011). On American campuses, female preponderance correlates with more hookups and sex partners, and an attitude toward copulation as something that does not require love (Adkins et al., 2015). Women’s subjective well-being is reduced (Richardson, 2021).

High-ratio environments motivate male prosociality. With fewer women available, men act more in line with what women value. If they have a mate, men are incentivized to invest greater care and resources in the pair-bond (Kokko and Jennions, 2008; Schacht and Borgerhoff Mulder, 2015). They are more inclined to marry and do so earlier in life (South and Trent, 1988). Women’s well-being increases without being offset by a reduction in overall male well-being, as an increase in marriage also benefits men (Richardson, 2021). Men are less violent, choosing instead to compete with other men economically (Guttentag and Secord, 1983; Pedersen, 1991; Barber, 2000, 2009; Maner and Ackerman, 2020)—although in some contexts, violence can increase (Filser et al., 2021; South et al., 2022). Men incline to entrepreneurship, saving, and working longer hours (Chang and Zhang, 2012). Husbands have higher wages (Angrist, 2002). Women benefit from more leisure, but also earn less prominent professional positions (Grosjean and Khattar, 2019). Bachelors spend more on dates (Griskevicius et al., 2012). Chinese parents save more when their sons face scarcity (Wei and Zhang, 2011). Men emphasize family and honoring women, and also virginity and monogamy to avoid competition from other men (Guttentag and Secord, 1983; Secord, 1983)—although they become more positive to sex tourism (Kock, 2021). Fewer children result from extrapair copulation (Schacht and Kramer, 2016), yet fertility increases (Schmitt and Rohde, 2013).

### 4.5. The future of pair-bonding

The above outcomes of a higher PSR contribute to why I propose that polygyny is worth reconsidering (Table 1). To what extent such mating would lead to high-ratio prosociality as opposed to the negative externalities that historically have been associated with polygyny, we cannot know beforehand. The twenty-first-century environment is so novel that it should inspire openness. Neither can we know to what extent polygyny can counter our era’s demographic collapse, but it should slow or reverse the current trend. Immigration has not stopped the decline in fertility; the practice has also become less culturally palatable in the West. The liberal universalism of the 1990s promoted that populations were more interchangeable than what the experiences of the past decades suggest. It seems unlikely that, for instance, Russia or China will permit large-scale immigration from African nations with high fertility to prop up their own population numbers. Western nations also seem increasingly unwilling to opt for such a solution.

**Table 1:** Consequences of polygyny.

**Potential downsides of polygyny:** zero-sum mindset from increased competition (Henrich, 2020); fewer mating opportunities for low-status men; reduced well-being for low-status men (Richardson, 2021); lower paternal investment; less economic growth as men pursue additional wives (Henrich, 2010); violence, crime, and social instability from young male syndrome (Blake and Brooks, 2022); too high fertility in some environments (Fenske, 2015); commoditization of women in patriarchal societies (Raffield et al., 2017a,b); male aggressive and high-risk behavior (Henrich, 2020); war when men seek to increase their pool of women (Gottschall, 2008); male mortality stratification (Bergløff, 2021).
**Potential upsides to polygyny:** increased fertility that counters demographic collapse (Henrich, 2010; Fenske, 2015); less divorce driven by serial monogamy; more children grow up in multiple-parent households; fewer single-person households and less loneliness; large households that inspire pride and belonging (Calder and Beaman, 2014); greater individual choice in pair-bonding decisions (Nussbaum, 2008; Mendelson, 2022); women get higher-status mates; fulfills high-status men’s desire for multiple partners (Chapais, 2011); reduced promiscuity; earlier pregnancy and longer reproductive periods (Henrich, 2020); higher quality offspring; less helicopter parenting (Lukianoff and Haidt, 2018).
**Potential outcomes of low PSR:** promiscuity; fewer and later marriages; more illegitimate children; dating deception; relationship instability (Secord, 1983; Pedersen, 1991; South, 1995; Schmitt, 2005; Schacht and Kramer, 2016); early pregnancy (South and Trent, 1988; Barber, 2000, 2001; Chipman and Morrison, 2013); reduced female well-being (Richardson, 2021); women raise mate standards; men reduce mate standards (Stone et al., 2007); sexual marginalization of low-status men (Almås et al., 2020; Ueda et al., 2020; Brooks et al., 2022); women permit more uncommitted sex; greater intra-female mate competition; women wear shorter skirts and post more sexualized selfies (Barber, 1999; Borgerhoff Mulder, 2018); greater male preference for short-term mating; some women marry down (Spanier and Glick, 1980); more singledom (Lichter et al., 1995; Bergløff, 2021; Fry and Parker, 2021; Yong et al., forthcoming); men date more; less cohabitation (Warner et al., 2011).
**Potential outcomes of high PSR:** increased fertility (Schmitt and Rohde, 2013); higher marriage rate; earlier and more stable marriages (South and Trent, 1988); greater male investment in pair-bonds (Kokko and Jennions, 2008; Schacht and Borgerhoff Mulder, 2015); women raise mate standards (Stone et al., 2007); women marry up (Albrecht et al., 1997; Pollet and Nettle, 2008); higher female well-being (Richardson, 2021); more male entrepreneurship; men work and save more (Wei and Zhang, 2011; Chang and Zhang, 2012); less male violence (Guttentag and Secord, 1983; Pedersen, 1991; Barber, 2000, 2009; Maner and Ackerman, 2020); men more motivated to pair-bond; women have more leisure; less gender-equal workplaces (Grosjean and Khattar, 2019); emphasis on family, virginity, monogamy, and honoring women (Guttentag and Secord, 1983; Secord, 1983); more sex tourism (Kock, 2021).

It is challenging to predict how polygynous practices would affect modern societies. This table sums up potential downsides and upsides. Permitting such pair-bonds would likely reduce singledom, which could contribute to a higher perceived sex ratio, that is, a sense of single women being scarcer. The table sums up potential outcomes of high and low PSRs.

Other recommendations are oddly absent, as is constructive debate (Eberstadt, 2021). Since a fear of overpopulation has marked the past generations, and climate change remains a grave threat, many find it challenging to respond to the surprising drop in fertility that many nations have experienced. We lack intuitions for how too rapid depopulation can threaten social and economic stability. That experts have so few solutions to offer disincentivizes broad debate. When even Norwegian social democracy fails to inspire women to reproduce—with a $1.2 million lifetime transfer (Statistics Norway, 2022b)—naturally other nations are unsure of which strings to pull on. Eastern Europe’s illiberal contestation of reproductive rights has not boosted fertility to any significant extent.

Bainbridge (2009) proposed that we prepare for a future as cyborgs. This may sound excessive, but others too wonder if conventional mating is losing out. Our present era’s Fourth Industrial Revolution is expected to add tremendous novelty to our already digitized mating markets (Brooks, 2021). Biological hacking, designer babies, and artificially intelligent sex robots will have hard-to-predict consequences (Harari, 2016). Musk (2014) fears that biological reproduction will no longer be adaptive, that humanity could end its days as “the biological boot loader for digital superintelligence.” One of his strategies for countering such a future, he keeps stating, is to set an example with prolific mating. Testifying to the attractiveness of reproduction with high-status men, Musk has, so far, fathered 10 children through serial monogamy and extrapair copulation. That 70% of Henrich’s female undergrads found being a billionairess so compelling (BCSC 1588, 2011)—in addition to the findings of this article—suggest that high-status men increasingly will be looked to for reproduction, whether polygynous pair-bonds are legalized or not. I am not convinced that permitting polygamy must be the best course of action. But considering the stakes we face, we owe ourselves and future generations a more imaginative debate around our era’s demographic collapse than what we have had so far.

## Data availability statement

The original contributions presented in the study are included in the article/supplementary material, further inquiries can be directed to the corresponding author.

## Author contributions

The author confirms being the sole contributor of this work and has approved it for publication.

## Conflict of interest

The author declares that the research was conducted in the absence of any commercial or financial relationships that could be construed as a potential conflict of interest.

## Publisher’s note

All claims expressed in this article are solely those of the authors and do not necessarily represent those of their affiliated organizations, or those of the publisher, the editors and the reviewers. Any product that may be evaluated in this article, or claim that may be made by its manufacturer, is not guaranteed or endorsed by the publisher.
